# Sensitive Detection of Gynecological Cancer Recurrence Using Circulating Tumor DNA and Digital PCR: A Comparative Study with Serum Biochemical Markers

**DOI:** 10.3390/ijms252211997

**Published:** 2024-11-08

**Authors:** Nour Balasan, Feras Kharrat, Giovanni Di Lorenzo, Emmanouil Athanasakis, Anna Monica Bianco, Andrea Conti, Maria Teresa Di Stazio, Giulia Butera, Stefania Cicogna, Alessandro Mangogna, Federico Romano, Giuseppe Ricci, Adamo Pio d’Adamo

**Affiliations:** 1 Institute for Maternal and Child Health-IRCCS “Burlo Garofolo”, 65/1 Via dell’Istria, 34137 Trieste, Italy; feras.kharrat@burlo.trieste.it (F.K.); emmanouil.a@gmail.com (E.A.); annamonica.bianco@burlo.trieste.it (A.M.B.); andrea.conti@burlo.trieste.it (A.C.); mariateresa.distazio@burlo.trieste.it (M.T.D.S.); giulia.butera@burlo.trieste.it (G.B.); stefania.cicogna@burlo.trieste.it (S.C.); alessandro.mangogna@burlo.trieste.it (A.M.); federico.romano@burlo.trieste.it (F.R.); giuseppe.ricci@burlo.trieste.it (G.R.); adamopio.dadamo@burlo.trieste.it (A.P.d.); 2 Department of Medicine, Surgery and Health Sciences, University of Trieste, Strada di Fiume 447, 34149 Trieste, Italy

**Keywords:** ctDNA, gynecological cancer, dPCR, biochemical markers

## Abstract

Early detection of recurrences in gynecological cancers is crucial for women’s health. Circulating tumor DNA (ctDNA) analysis through liquid biopsy offers a promising approach for monitoring disease progression and identifying relapses. This study investigated the utility of digital Polymerase Chain Reaction (dPCR) for ctDNA detection in three gynecological cancer patients with clinically confirmed relapses during a two-year post-surgical follow-up. Patient-specific tumor mutations were identified through whole-exome sequencing (WES) and confirmed via Sanger sequencing. dPCR probes targeting these mutations were used to quantify the ctDNA levels in plasma samples collected throughout the follow-up period, and the findings were compared with standard serum biochemical markers. In two patients, persistent positive dPCR signals for the selected mutations were detected after tumor removal, with ctDNA levels progressively increasing even after post-surgical chemotherapy. Notably, dPCR identified elevated ctDNA levels before an increase in the cancer antigen 125 (CA125) biochemical marker was observed. In the third patient, no ctDNA signals from the two selected mutations were detected despite clinical evidence of recurrence, suggesting the emergence of new mutations. While this study highlights the promise of dPCR for early recurrence detection in gynecological cancers, it also underscores the critical need for comprehensive mutation panels to overcome the inherent challenges posed by tumor heterogeneity and the emergence of new mutations during disease progression.

## 1. Introduction

Gynecological tumors comprise a diverse group of malignancies primarily affecting the uterus, ovaries, and cervix, with less frequent occurrences in the vulva and vagina. In higher-income countries, endometrial cancer is the most prevalent gynecological malignancy, prevalent in post-menopausal women. Cervical cancer, the fourth most frequently diagnosed cancer among women of reproductive age globally, follows breast, lung, and colorectal cancers in prevalence [1]. Ovarian cancer, often diagnosed at advanced stages [2], predominantly manifests as high-grade serous carcinoma [3]. Recurrence rates vary across gynecological cancers, but many relapses occur within the first three years post-treatment [4].

The recurrence of gynecological tumors presents significant clinical challenges, often leading to poor prognosis. Early detection is paramount for effective recurrence management. Current clinical practice relies on serum biochemical tumor markers, such as CA125, to monitor cancer patients. However, these markers often lack the sensitivity and specificity required for reliable early detection [5,6].

Tumor cells shed DNA fragments, known as circulating tumor DNA (ctDNA), into the bloodstream. These fragments harbor tumor-specific mutations (somatic), making them valuable biomarkers for disease detection and monitoring. The percentage of ctDNA in the bloodstream varies widely, influenced by factors such as cancer stage and type, tumor size and aggressiveness, and overall tumor burden [7,8].

Many studies unraveled the potential use of the circulating tumor DNA as a prognostic tool for different types of cancers, including ovarian cancer [9,10,11,12,13] and endometrial cancer [14,15].

In another study by Recio et al. [16], it was reported that ctDNA monitoring, besides other risk factors, can be beneficial in identifying patients with stage I endometrial cancer who are at elevated risk of recurrence, and might guide the treatment stratification. Another study reported that ctDNA could be used to evaluate the treatment efficiency of gynecologic cancer patients [17].

Liquid biopsy, a non-invasive technique for analyzing ctDNA, offers insights into these tumor-specific mutations. Many liquid biopsy strategies for early relapse detection utilize panels targeting specific known mutations, often found in genes commonly associated with various cancer types [18]. These panels focus on well-characterized driver mutations. Examples include the FDA-approved Guardant360 CDx and FoundationOne Liquid CDx tests, which are designed for patients with specific lung and prostate cancer types [19,20]. However, these pre-defined mutations may not always align with the somatic mutations present in individual patients’ tumor DNA, limiting the applicability of liquid biopsy for some individuals. This underscores the importance of developing personalized tests to overcome the limitations of currently available panels.

The limitations of pre-defined mutation panels in liquid biopsies have spurred advancements in molecular diagnostics. Approximately 35 years after Kawasaki’s groundbreaking use of PCR in cancer diagnostics to detect chronic myelogenous leukemia cells [21], the field has evolved significantly. The advent of digital PCR, coupled with the rise in liquid biopsies for analyzing circulating cell-free DNA (cfDNA), has revolutionized the landscape of cancer detection and monitoring.

dPCR stands out as a powerful technique for detecting and quantifying ctDNA. Its high accuracy, sensitivity, reproducibility, and capacity for absolute quantification make it increasingly valuable in liquid biopsies for personalized cancer management [22].

In the present study, we harnessed the capabilities of dPCR and liquid biopsy to identify patient-specific ctDNA mutations associated with gynecological tumors. By analyzing plasma samples, we aimed to differentiate ctDNA from the broader pool of cfDNA.

This approach offers a promising avenue for personalized, non-invasive early cancer detection, addressing the limitations of pre-defined panels and potentially reducing the need for invasive procedures.

## 2. Results

### 2.1. Patient 1

Patient 1 underwent surgery for uterine bleeding in post-menopause in October 2020, and the final histopathologic diagnosis of endometrial biopsy was endometrioid carcinosarcoma G3 and chondrosarcoma, classified as stage IB according to the International Federation of Gynecology and Obstetrics (FIGO) 2009 classification.

A commercial dPCR probe targeting a somatic single nucleotide variant (SNV) in the *TP53* gene (*TP53*:c.734G>T:p.G245V), identified through whole-exome sequencing of the tumor and absent in genomic DNA, was chosen to monitor early recurrence during the post-surgical follow-up. In our WES analysis of the patient’s tumor tissue, this mutation’s allele frequency was 0.823. When this specialized probe was first tested with the patient’s tumor DNA and normal genomic DNA, it detected the mutation in 81.5% of the tumor DNA and not at all in the genomic DNA. Then, five plasma samples were tested (Table S1).

**Figure 1:**
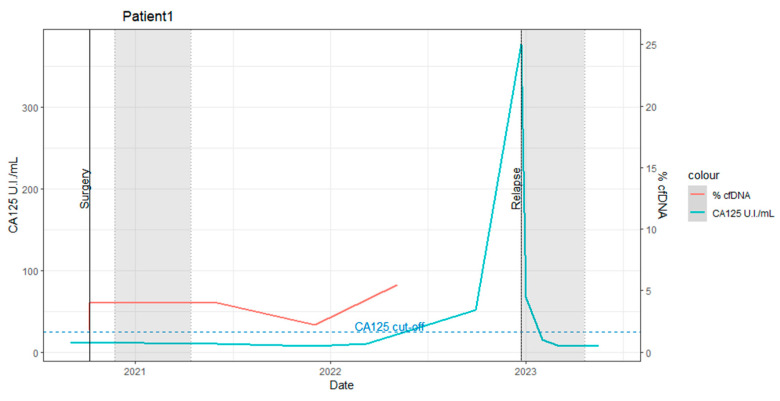
Time course of CA125 elevated levels and ctDNA percentages in patient 1 during the study. The horizontal axes indicate the date, and the left vertical axes indicate the score of CA125 (unit/mL: blue line). The ctDNA percentage is shown with the right vertical axis (%: red line). The gray zones indicate the period of adjuvant therapy. The black vertical lines indicate clinical events such as surgical resection or relapse detection.

- Pre-surgery: The baseline levels of biochemical markers were within normal ranges. The percentage of ctDNA carrying the *TP53* SNV in the plasma sample was 1.8%.
- Post-surgery: The ctDNA percentage slightly increased to 4%, suggesting incomplete tumoral removal or metastasis. This underscores the utility of dPCR in estimating the efficacy of surgical interventions. The patient subsequently underwent 5 months of adjuvant chemo-radiotherapy (the first gray zone in Figure 1).
- Two months post-adjuvant therapy: The ctDNA percentage remained stable at 4%.
- Eleven months post-adjuvant therapy: The ctDNA percentage increased to 5.5% at 11 months (as indicated in Figure 1, (ctDNA%: red line)).
- Eighteen months post-adjuvant therapy: A slight elevation in CA125 (51.49 UI/mL) was observed without clinical evidence of disease progression.
- Nineteen months post-adjuvant therapy: Clinical recurrence was detected via a positron emission tomography (PET)-computed tomography (CT) scan.

dPCR analysis revealed minimal residual disease or metastasis immediately after surgical tumor resection, with a gradual increase in the ctDNA percentage during the follow-up.

Unfortunately, the patient decided to withdraw from our study after eleven months post-adjuvant therapy. As a result, we were unable to collect further ctDNA% data beyond this timepoint.

Note: Detailed clinical information is provided in Supplementary Material S1. Absolute quantification dPCR results with corresponding follow-up sampling times are presented in Table S1.

### 2.2. Patient 2

Patient 2 was diagnosed with high-grade serous ovarian cancer (HGSOC) G3, stage IIB (FIGO 2014), after a staging laparoscopy (S-LPS) performed in January 2020. A somatic *PIK3CA:*c.3140A>G:p.H1047R mutation with a high allele frequency in our WES analysis (0.9), identified through whole-exome sequencing of the tumor and absent in genomic DNA, was selected as a marker for early recurrence monitoring. A commercial dPCR probe targeting this specific mutation was employed. Upon primary testing using a specific commercial dPCR probe, the presence of the *PIK3CA* mutation in tumor DNA was detected at a rate of 86.1%, whereas in genomic DNA, the mutation was detected at a rate of 0.03%. For follow-up, five plasma samples were tested (Table S2).

**Figure 2:**
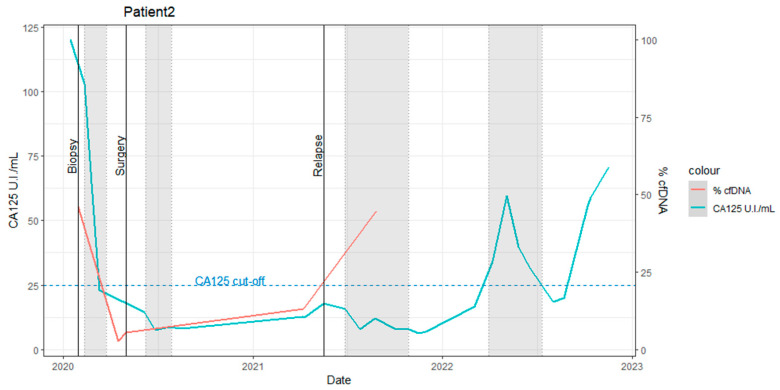
Time course of CA125 elevated levels and ctDNA percentages in patient 2 during the study. The horizontal axes indicate the date, and the left vertical axes indicate the score of CA125 (unit/mL: blue line). The ctDNA percentage is shown with the right vertical axis (%: red line). The gray zones indicate the period of adjuvant therapy. The black vertical lines indicate clinical events such as surgical resection or relapse detection.

- Pre-staging laparoscopy (S-LPS): The patient presented with elevated CA125 levels (120 UI/mL).
- Post-staging laparoscopy (S-LPS): A high ctDNA percentage (~46%) was detected (Figure 2, ctDNA%; red line). The patient underwent three cycles of neoadjuvant chemotherapy (the first gray zone in Figure 2).
- Post-chemotherapy: Minimal residual disease was detected in plasma (2.7% ctDNA), with a concomitant decrease in CA125 levels. Laparotomic interval debulking surgery (IDS), performed in April 2020, revealed a 5 mm macroscopic residual lesion, consistent with the ctDNA finding.
- Post-IDS: Despite normalized CA125 levels, the persistence and increase in the *PIK3CA* mutation (2.7% to 5.6% ctDNA) suggested a potentially incomplete surgical resection. The patient received three additional cycles of adjuvant chemotherapy.
- Eight months post-chemotherapy and IDS: The ctDNA percentage increased to 13%, indicating potential chemotherapy resistance. Thus, in this case, a change in medication is advisable for the subsequent cycle.
- Ten months post-chemotherapy and IDS: Recurrence was detected via a contrast-enhanced chest-abdomen CT scan.
- Thirteen months post-chemotherapy and IDS: The ctDNA percentage further increased to ~45%.
- Eleven months post-recurrence detection: CA125 levels began to rise.

These findings are summarized in Figure 2. Detailed clinical information is provided in Supplementary Material S2, and absolute quantification dPCR results with corresponding follow-up sampling times are presented in Table S2.

### 2.3. Patient 3

Patient 3 underwent S-LPS in July 2020. The final histopathologic diagnosis of the tumor biopsy was advanced HGSOC grade G3, stage IIIC. Two custom dPCR probes were designed to target two somatic mutations, one in the *CEP135* gene (*CEP135*:c.3389C>T:p.S1130F) and the other in the *CCNF* gene (*CCNF*:c.541-31G>C), identified through whole-exome sequencing of the tumor and absent in genomic DNA. The allele frequencies of these mutations were determined in our analysis to be 0.246 and 0.283, respectively.

**Figure 3:**
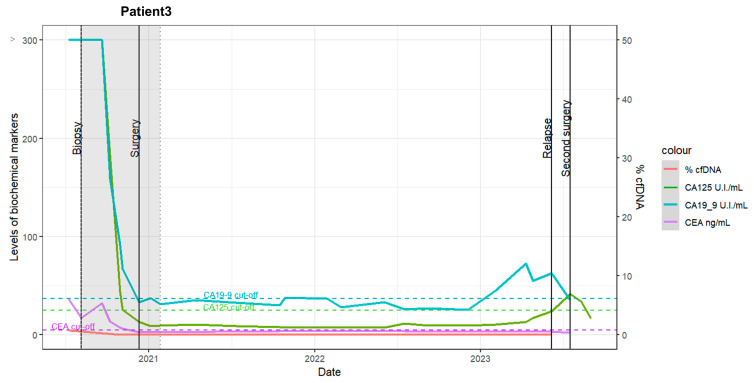
Time course of elevated levels of biochemical markers (CA125, CA19-9, and CEA) alongside the ctDNA percentages in patient 3. The date is represented on the horizontal axes, while the scores of biochemical markers are shown on the left vertical axes. The CA125 levels are drawn by the green line (unit/mL), CA19-9 levels by the blue line (unit/mL), and CEA levels by the purple line (ng/mL). The CtDNA percentage is indicated on the right vertical axis (%: red line). The gray zone indicates the period of chemotherapy, and the perpendicular lines denote significant clinical events, such as surgical resection or relapse detection.

- Pre- and post-staging laparoscopy (S-LPS): The patient initially underwent S-LPS, followed by four cycles of neoadjuvant chemotherapy. Elevated cancer antigen 125 (CA125) and carbohydrate antigen 19-9 (CA19-9) marker levels were observed pre-chemotherapy, with minimal ctDNA detected for both targets (<1%) before and after S-LPS (Figure 3, red line).
- Post-chemotherapy: A marked decrease in biochemical marker levels was observed following chemotherapy.
- Laparotomic interval debulking surgery (IDS) and adjuvant chemotherapy: The patient underwent laparotomic IDS and four additional chemotherapy cycles.
- Recurrence: Tumor recurrence was detected via a positron emission tomography-computed tomography (PET-CT) scan 27 months after the completion of chemotherapy.

Using tumor biopsy-derived tumor DNA as a positive control in dPCR, we observed mutation prevalence rates of 33% for the *CEP135* gene and 34% for the *CCNF* gene. When genomic DNA was used as a negative control, mutation rates of 0% were observed for both mutations, as indicated in the absolute quantification dPCR results in Table S3 (absolute quantification dPCR result of the mutation in the *CEP135* gene) and Table S4 (absolute quantification dPCR result of the mutation in the *CCNF* gene). However, according to the two probes, in 11 cfDNA samples collected during the follow-up, few positive signals were detected for both pre- and post-biopsy surgery. Meanwhile, no detectable ctDNA signals were detected when nine subsequent follow-up samples were collected post-S-LPS (as indicated by the red line in Figure 3). From a clinical standpoint, CA125 and CA19-9 marker levels were initially high before decreasing during the following chemotherapy treatment.

Detailed clinical information is available in Supplementary Material S3.

## 3. Discussion

Early detection of gynecological tumor recurrences is crucial for women’s health, particularly in younger patients where timely intervention can improve disease management and potentially preserve fertility. Liquid biopsy, with its ability to analyze circulating tumor DNA (ctDNA), holds great promise as a reliable and non-invasive diagnostic tool for monitoring disease progression and identifying relapses in gynecological cancers. ctDNA provides valuable insights into tumor characteristics and can inform personalized treatment strategies. Emerging omics technologies, coupled with the high sensitivity of digital PCR, have facilitated the development of customized assays to track tumor evolution. This is particularly important for aggressive cancers with high recurrence rates, such as ovarian cancer.

In this study, we analyzed ctDNA in plasma samples from patients with gynecological malignancies during a post-treatment follow-up. Somatic mutations, previously identified through exome sequencing of tumor tissue DNA, were targeted using patient-specific dPCR probes in three patients who experienced recurrence. Mutated ctDNA fragments were detected in plasma samples from two of these patients during the two-year follow-up period. We selected for patient 1 a pathogenic somatic mutation (*TP53*:G245V). This mutation is a hotspot mutation that lies in the DNA binding site of the *TP53* protein [23], and is shown to decrease the *TP53* protein’s growth suppression ability [24]. The mutation was reported in the COSMIC (Catalogue Of Somatic Mutations in Cancer) database as a drug-resistance mutation and reported in the literature as a hotspot-predicted prognosis mutation in other types of cancers, such as lung cancer [25] and gastric cancer [26]. The tDNA percentage for the mutation in the *TP53* gene was 81% (as shown in the Results), indicating that most of the circulating tumor cells harbor this mutation. During the follow-up, this mutation was used as an indicator for the recurrence.

For patient 2, the *PIK3CA*: H1047R actionable mutation was selected. The tDNA percentage for this mutation was 86%, indicating the high prevalence of this mutation in the tumor cells. This mutation is a hotspot mutation within the kinase domain of the *PIK3CA* protein, and results in the increased phosphorylation of *AKT* and *MEK1/2* to enhance growth factor signaling and cell survival [27,28].

Regarding patient 3, the WES did not detect any reported known cancer-driver mutations, so we analyzed the ctDNA levels of two somatic mutations in the *CCNF* and *CEP135* genes, and custom-designed probes were used. The tDNA percentages for these mutations by dPCR were 34% for *CCNF* and 33% for *CEP135*, which were lower than the values of the tested mutations for the first two patients. This suggests that chemotherapy treatment post-S-LPS likely led to an effective response, resulting in decreased biochemical marker levels and potentially eliminating tumor cells harboring these specific mutations. It is plausible that the genetic profile of the secondary tumor differs from that of the primary one.

Several studies have highlighted the utility of droplet digital PCR (ddPCR) for detecting relapses in gynecological tumors. For example, Minato et al. reported that when using ddPCR to detect ctDNA from ovarian cancer patients with recurrences, the recurrence was detected earlier than the normal method based on CA125 [29]. In another study by ddPCR, ctDNA levels were measured in 60 endometrial carcinoma (EC) patients, and it was reported that 56.3% of patients with high-risk tumors were positive for ctDNA at the time of the surgery, while the percentage of patients with low-risk tumors was 15.8%. The same study reported higher levels of ctDNA in patients with high tumor grade, indicating the utility of ctDNA to monitor the tumor burden [15]. The correlation between the ctDNA levels and grade, stage, and histopathological type was also reported by another study using the ddPCR by targeting mutations in the *PIK3CA* or *KRAS* genes in patients with EC [30]. An important point to be highlighted for the utility of ctDNA is the sample timing. In a study by Feng et al. [31], the postoperative but not preoperative ctDNA levels were shown to predict tumor recurrence in EC patients. Interestingly, the same study reported that ctDNA was significantly correlated with only FIGO classification and lymph node status among the clinicopathological findings.

To our knowledge, this is the first study to employ nanoplate dPCR for investigating recurrences using patient-specific somatic mutations. A PubMed search using the keyword “Nanoplate digital PCR” yielded no results related to gynecological tumors, underscoring the novelty of our approach.

While dPCR offers a high potential for relapse detection, this innovative technology must be used carefully. As shown in the results, by applying the dPCR method, we were able to detect the recurrence earlier than other clinical methods in the first and second patients, but in patient 3, dPCR yielded negative results for both tested mutations, despite clinical evidence of recurrence. This discrepancy may be attributed to the accumulation of additional mutations in advanced-stage tumors, potentially affecting primer annealing and hindering mutation detection.

These findings underscore the importance of targeting multiple mutations simultaneously to capture a more comprehensive snapshot of tumor heterogeneity and enhance the accuracy of relapse detection. We recommend analyzing at least five different mutations with relatively high allele frequencies within the tumor mass, as this reflects their presence in the majority of tumor clones and may mitigate the risk of false negatives in ctDNA-based monitoring. The study protocol involved collecting 5 mL of plasma during follow-up visits. Given the typically low concentration of cfDNA in plasma samples, this volume effectively limited the number of mutations we could reliably test. Based on this experience, we recommended to collect 10 mL of plasma in future studies, which would enable the testing of additional mutations and thus provide more comprehensive molecular monitoring.

Finally, this study did not calculate sensitivity, specificity, the positive predictive value (PPV), and the negative predictive value (NPV) for our method. These metrics require a long-term follow-up to identify false negatives, which are determined by whether recurrence occurs after a negative test result. Due to the limited number of recurrences and the study’s short duration, a reliable establishment of these values was not feasible.

This limitation will be addressed in future research with a larger cohort and extended follow-up. The current study serves as a proof-of-concept of utilizing these technologies, demonstrating that in cases where recurrence did occur, our approach would have facilitated significantly earlier detection compared to the current standard methods.

## 4. Materials and Methods

### 4.1. Patient Enrolment

Between 3 June 2019 and 11 April 2022, 36 patients diagnosed with various gynecological tumors (including endometrial, ovarian, and cervical cancers) in FIGO stages I–III were enrolled at the Institute for Maternal and Child Health-IRCCS “Burlo Garofolo” (Trieste, Italy). The study was approved and funded by the Institute’s Internal Review Board (approval number: Prot. 0026500/P/GEN/ARCS).

All enrolled patients provided written informed consent for the collection of clinical data and biological samples.

Patient medical records were reviewed to gather information on demographics, initial tumor site, histological characteristics, tumor grade and stage, levels of specific tumor markers (collected every three months during follow-up), and details of chemotherapy regimens. Additionally, data on surgical outcomes, sites of metastasis, and instances of tumor recurrence were compiled.

Whole-exome sequencing (WES) was performed on tumor samples from 36 patients who had at least four plasma samples collected during follow-up. Among these, 9 patients withdrew from the study, and only 3 experienced a recurrence. Our analysis focused on these 3 patients because they experienced a recurrence within two years post-surgery and underwent standard monitoring.

### 4.2. Sample Collection and DNA Extraction

**Figure 4:**
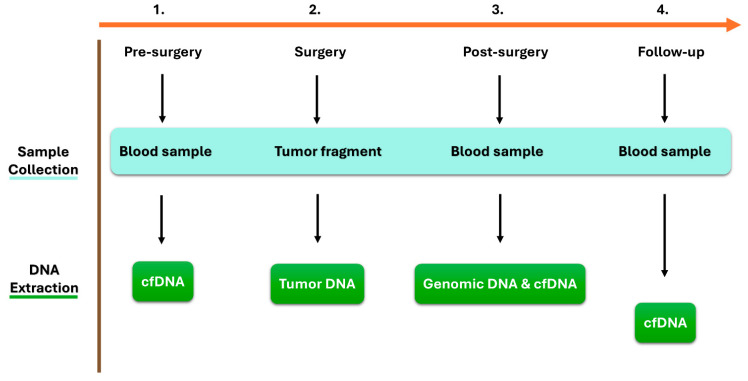
Sample collection and DNA extraction.

The following biological samples were collected for each enrolled patient (Figure 4):

- Whole blood samples pre-surgery, forty-eight hours post-surgery, and during the follow-up period every three months were collected using QIAGEN PAXgene^®^ Blood ccfDNA Tubes (catalog number: 768165, Becton, NJ, USA). The blood was centrifuged at 1900× *g* 4 °C for 10 min, and the supernatant plasma was transferred to separate Eppendorf Tubes. The plasma was further centrifuged at 1900× *g* 4 °C for 10 min to remove the remaining leukocytes. The obtained plasma samples (5 mL) were frozen and stored at −80 °C until the purification of circulating cell-free DNA (ccfDNA). The ccfDNA was extracted from 5 mL of plasma using the QIAGEN QIAamp^®^ DSP Circulating NA Kit (catalog number: 61504, Hilden, Germany), according to the manufacturer’s instructions.
- Genomic DNA (gDNA) was extracted from an aliquot of blood collected post-surgery in an EDTA tube. gDNA was extracted using the QIAGEN QIAamp^®^ DNA Blood Mini Kit (catalog number: 51104, Hilden, Germany), according to the manufacturer’s instructions, and used as healthy DNA.
- Tumor biopsies were collected at the time of initial surgery. Surgically removed cancer tissues were macroscopically selected based on the corresponding hematoxylin-eosin staining. The obtained tumor biopsies were stored at −80 °C until the extraction of tumor DNA. Tumor DNA was then extracted using the salting-out extraction procedure described by Miller et al. [32].

### 4.3. Whole-Exome Sequencing and Bioinformatic Analysis

The matched tumor DNA and genomic DNA samples of each patient were subjected to whole-exome sequencing (WES). First, 1 μg of each sample was sent to the Macrogen company (Seoul, Republic of Korea), the library was prepared using the Agilent (Twist Human Core Exome (+RefSeq)), and WES was performed using the NovaSeq™ 6000 sequencing platform by Illumina (Illumina Inc., San Diego, CA, USA). The bioinformatics procedures are summarized in Supplementary Figure S1. Quality assessment of the raw NGS data was performed using FastQC (https://www.bioinformatics.babraham.ac.uk/projects/fastqc/, accessed on 31 May 2023) [33]. Data pre-processing and variant calling were conducted with GATK v4.1. (https://gatk.broadinstitute.org/, accessed on 31 May 2023) [34] (Supplementary Figure S1, Step.1,2). The sequence was mapped to Human Genome GRCh38. All variants were annotated using the Annovar tool (https://annovar.openbioinformatics.org/en/latest/, accessed on 31 May 2023) [35], using ready-to-use and customized databases, such as the gnomAD (Genome Aggregation Database) for germline mutations, COSMIC (Catalogue Of Somatic Mutations in Cancer), TCGA (The Cancer Genome Atlas), and ICGC (International Cancer Genome Consortium), for somatic mutations. (Supplementary Figure S1, Step.3). All clinical data and identified variants were stored in a custom database created with pgAdmin4 (https://www.pgadmin.org/, accessed on 31 May 2023) [36] (Supplementary Figure S2).

Variant prioritization was carried out using the pgAdmin relational database by PostgreSQL to select some patient-specific tumor mutations that were subsequently used to check relapses. Potentially somatic mutations were selected through a filter, as described in Supplementary Figure S2. Some filters were applied to detect common mutations and keep only the mutations present in tumor DNA that are highly likely to be somatic. Further filtering conditions were applied for the output of the previous filter as follows: variants with an allelic frequency ≥ 0.2; minimum depth ≥ 100; and variants that were reported in the ICGC database and the COSMIC database were included. While patient variants marked as “germline” by the Mutect2 tool during variant calling or marked as “germline” were reported in the gnomAD database, they were excluded (https://gatk.broadinstitute.org/hc/en-us/articles/360037593851-Mutect2, accessed on 31 May 2023).

### 4.4. Validation of the Data by Sanger Sequencing

Sanger sequencing was carried out to validate potential somatic variants obtained from the variant prioritization step. Wild-type DNA and tumor DNA from the same patient were subjected to Sanger sequencing for each target. First, primers were designed flanking the variation ~250 bp upstream and ~250 bp downstream using the OligoAnalyzer™ tool by IDT™ (https://eu.idtdna.com/pages/tools/oligoanalyzer, accessed on 31 May 2023). Second, PCR amplification of the regions was performed under standard PCR conditions with annealing temperature in the range between 54 and 64 °C, followed by PCR product purification using ExoProStar^TM^ 1-Step, Enzymatic PCR, and the Sequence Reaction Clean-up kit. Subsequently, the samples were sent to the Macrogen company for sequencing. Sanger sequencing data were downloaded from the Macrogen company’s server and electropherograms were visualized using the free tool Chromas Lite. A potentially somatic variant was considered a true somatic variant if it was present in the tumor DNA and absent in the wild-type DNA of the same patient.

### 4.5. Digital PCR

QIAGEN dPCR Mutation Assays were used to select commercially available probes for somatic mutation detection. Two specific dPCR LNA™ Mutation Assays (200) were purchased for two different patients from the QIAGEN company (catalog number: 250200, Hilden, Germany): 

For patient 1, we selected a probe (ID: DMH0000375 dPCR Mutation Assay *TP53* 11196 Human COSV52666323) that targets a somatic mutation in the *TP53* gene (NM_000546:exon7:c.734G>T:p.G245V).

For patient 2, we chose a probe (ID: DMH0000036 dPCR Mutation Assay *PIK3CA* 775 Human COSV55873195) for a somatic mutation in the *PIK3CA* gene (NM_006218:exon21:c.3140A>G:p.H1047R).

And for patient 3, two Custom dPCR LNA Assays were designed and tested by the QIAGEN company. The first probe targets a somatic mutation in the *CEP135* gene (NM_025009:exon25:c.3389C>T:p.S1130F) and the second is for a mutation in the *CCNF* gene (NM_001761:exon6:c.541-31G>C).

The probe fluorophores were FAM (green)/HEX (yellow). The probe efficiency was tested initially with DNA extracted from the corresponding tumor (mutant) and blood (genomic DNA/wild-type) specimens for all used probes.

PCR reactions were prepared according to the provided protocol: For each reaction, we used two different controls in order to compare our findings during the follow-up with them to check the relapse: the first control is the gDNA/tDNA of the patient as the negative/positive control, respectively. The second control is by using the cfDNA extracted from the samples collected before surgery (positive control) and post-surgery (negative control) to check if the tumor mass was completely removed by the surgery procedure.

For the cfDNA samples: 10 μL of eluted cfDNA (~0.30–0.50 μg), 10 μL of 4× Probe PCR Master Mix (Qiagen), 4 μL of 10× primer-probe mix, and 16 μL of DNase/RNase-free water (total volume: 40 μL). For the gDNA/tDNA samples that were used as controls, 1 μL of tDNA/gDNA (~0.50 μg) with 1 μL (~0.25 U/μL) of the EcoRI restriction enzyme were added to the reaction mix (enzymatic fragmentation of larger DNA (≥20 kb average length) is recommended in order to distribute the template throughout the QIAcuity Nanoplate). DNase/RNase-free water was added to achieve a final volume of 40 μL, It is important to note that by decreasing gDNA/tDNA concentrations, we can avoid the saturated signals and create a balance between these samples and the cfDNA samples that were loaded in the same plate.

The contents of each well were then transferred into the wells of a QIAcuity^TM^ Nanoplate 26 k 24-well nanoplate (catalog number: 250001, Hilden, Germany), in which one reaction mix is separated into approximately 26,000 partitions. The nanoplate was sealed using the QIAcuity Nanoplate seal provided in the QIAcuity Nanoplate Kit. The PCR conditions were as follows: one cycle at 95 °C for 2 min, 40 cycle steps at 95 °C for 15 s, and 60 °C for 30 s. After PCR, the amplification target was identified by assessing the fluorescence in all positive partitions. Poisson statistics were employed to determine the average quantity of target DNA per partition. The total amount of target DNA in all partitions within each well was computed by multiplying the average target DNA per partition with the number of valid partitions. QIAcuity Software Suite 2.1.8.23 analyzes the mutation frequencies in target samples as the absolute quantification of copies of each wild-type and mutant target present in the samples. The percentage of mutant positive partitions was calculated for each sample manually at the end of the reaction for the mutated and wild-type signals.

ctDNA (%) = (number of positive partitions of mutant signal/total number of positive partitions from both signals) × 100.

## 5. Conclusions

The detection of minimal residual disease (MRD) in gynecologic cancers, as in other malignancies, poses a significant diagnostic challenge and represents a critical opportunity for precision medicine. Our study highlights the potential of personalized ctDNA biomarkers in gynecologic cancers, demonstrating their utility in detecting residual tumor cells and offering a more precise and dynamic assessment of treatment response compared to conventional methods such as serum tests and imaging studies. Early identification of disease persistence through ctDNA monitoring could enable prompt therapeutic interventions, potentially improving patient outcomes and survival rates.

From a clinical perspective, confirming relapse in a patient necessitates a multi-marker approach.

However, further research and clinical validation are imperative to establish the clinical utility and optimal implementation of ctDNA-based monitoring in routine practice, ultimately leading to improved patient management and prognosis.

## Data Availability

The data presented in this study are available on request from the corresponding author. The data are not publicly available due to ethical reasons.

## Supplementary Materials

The following supporting information can be downloaded at: https://www.mdpi.com/article/10.3390/ijms252211997/s1. Reference [37] is cited in supplementary materials.
