# Supplementary material for: Sensitive Detection of Gynecological Cancer Recurrence Using Circulating Tumor DNA and Digital PCR: A Comparative Study with Serum Biochemical Markers
Source: Int J Mol Sci. 2024 Nov 8;25(22):11997. doi: 10.3390/ijms252211997 (PMC11593349; doi:10.3390/ijms252211997)
Supplement: Supplementary file 1 [file ijms-25-11997-s001.zip › ijms-3263111-supplementary.pdf]

International Journal of *Molecular Sciences*

MDPI

Supplementary Material

The following is the Supplementary Material to this article:

Supplementary Material S1

Patient 1: A 76-year-old woman was diagnosed with endometrial carcinosarcoma. After endometrial biopsy performed for uterine bleeding in post-menopause, she underwent laparoscopic radical hysterectomy, bilateral salpingo-oophorectomy, sentinel lymph node mapping, and peritoneal washing, with no macroscopic residual disease in October 2020. The final histopathologic diagnosis was endometrioid carcinoma G3 and chondrosarcoma, stage IB (FIGO 2009).

Before surgery, the serum levels of biochemical markers (CA125, CEA, CA19-9, AFP, beta-HCG) were normal. Due to the high-risk histopathology, 48 days post-surgery procedure, she underwent adjuvant chemo-radiotherapy until April 2021 (two cycles of chemotherapy with Carboplatin AUC5, then external beam radiation therapy (EBRT) 45 gray (Gy) and brachytherapy 10 Gy, followed by two additional cycles of Carboplatin AUC5).

The patient relapsed 19 months post-adjuvant therapy (peritoneal carcinomatosis detected by PET-CT scan in November 2022). Throughout adjuvant treatment and the subsequent follow-up, the levels of the CEA, AFP, CA19-9, beta HCG, and CA125 markers remained within normal ranges until September 2022, where a little rise in CA125 was observed (CA125 51.49 UI/mL), without clinical evidence of the progression of the disease until November 2022.

Table S1. Absolute quantification of dPCR analysis for the plasma samples collected during the follow-up with the gDNA and tDNA for patient 1. The mutated probe of the specific mutation in TP53 is indicated with green fluorescent dye and the wild type is indicated with yellow dye.

Well	Sample/NTC/Control	Type	Reaction Mix	Channel (Name)	Conc. [cp/µL] (dPCR Reaction)	CI (95%) (dPCR Reaction)	Partitions (Valid)	Partitions (Positive)	Partitions (Negative)	Threshold
D1	gDNA	Sample	TP53	GREEN	0	-	25,454	0	25,454	46.16
		Sample	TP53	YELLOW	444.5	0.023	25,328	7074	18,254	58.65
D2	tDNA	Sample	TP53	GREEN	106.3	0.046	25,475	1839	23,636	53.55
		Sample	TP53	YELLOW	23.41	0.096	25,475	417	25,058	56.1
D3	Pre-surgery	Sample	TP53	GREEN	1.525	0.37	25,458	28	25,430	37.23
		Sample	TP53	YELLOW	84.39	0.051	25,446	1503	23,943	51.32
E1	Post-surgery	Sample	TP53	GREEN	5.76	0.189	25,454	107	25,347	60.56
		Sample	TP53	YELLOW	141.6	0.039	25,440	2503	22,937	59.29
E2	2 months post-adjuvant therapy	Sample	TP53	GREEN	2.266	0.306	25,466	41	25,425	37.87
		Sample	TP53	YELLOW	55.54	0.062	25,462	986	24,476	56.74
E3	8 months post-adjuvant therapy	Sample	TP53	GREEN	1.187	0.418	25,461	22	25,439	34.68
		Sample	TP53	YELLOW	52.88	0.063	25,461	962	24,499	49.09
F1	11 months post-adjuvant therapy	Sample	TP53	GREEN	3.376	0.247	25,453	63	25,390	63.75
		Sample	TP53	YELLOW	59.59	0.059	25,449	1089	24,360	58.33

Supplementary Material S2

Patient 2: A 75-year-old woman was diagnosed with advanced high-grade serous ovarian cancer G3, stage IIB (FIGO 2014), in January 2020. After a staging laparoscopy (S-LPS), to assess the extent of the disease according to the Fagotti predictive index [PIV] value [37], a primary debulking surgery (PDS) was found to be unfeasible due to the high

burden of peritoneal disease. After S-LPS, the patient exhibited elevated CA125 levels (CA125=103 U/mL).

In February 2020, she underwent neoadjuvant chemotherapy (NACT) with three cycles of Carboplatin AUC5 / Paclitaxel 175 mg/m2 every 3 weeks, with a radiological and biochemical partial response.

The patient then underwent laparotomic interval debulking surgery (IDS) with radical hysterectomy, bilateral salpingo-oophorectomy, ileal resection, sigmoid-rectal resection, ileal–ileal anastomosis, ileostomy, removal of bulky lymph nodes, and removal of peritoneal carcinomatosis nodules. Interestingly, macroscopic residual disease of 5 mm was observed, which confirmed our findings about the ctDNA percentage (2.7%).

After IDS, CA125 levels were normal, but the presence of the mutation with an increase (from 2.7% to 5.6%) indicated a potentially incomplete surgical intervention. After that, she completed adjuvant chemotherapy with three cycles of Carboplatin AUC5 / Paclitaxel 175 mg/m2 every 3 weeks, with the last cycle occurring in July 2020. The patient relapsed 10 months after the last cycle of first-line chemotherapy (pelvic and para-aortic lymph nodal enlargement detected by CT scan in May 2021) via contrast-enhanced chest-abdomen CT. Meanwhile, CA125 levels began rising 11 months after clinical recurrence detection.

In June 2021, she was subjected to a second line of chemotherapy with Carboplatin AUC5 every 3 weeks, in consideration of partial platinum sensitivity (platinum-free interval of 10 months). Meanwhile, the ctDNA percentage increased more (~45%) in August 2021. Unfortunately, data after August 2021 are unavailable as the patient was no longer part of our study.

Table S2. Absolute quantification of dPCR analysis for the plasma samples collected during the follow-up with the gDNA and tDNA for patient 2. The mutated probe of the specific mutation in PIK3CA is indicated with green fluorescent dye and the wild type is indicated with yellow dye.

Well	Sample/NTC/Control	Type	Reaction Mix	Channel (Name)	Conc. [cp/µL] (dPCR Reaction)	CI (95%) (dPCR Reaction)	Partitions (Valid)	Partitions (Positive)	Partitions (Negative)	Threshold
A1	gDNA	Sample	PIK3CA	GREEN	0.104	1.19	25,426	2	25,424	57.63
		Sample	PIK3CA	YELLOW	364.9	0.025	25,197	6078	19,119	57.69
A2	tDNA	Sample	PIK3CA	GREEN	357.7	0.025	25,364	5970	19,394	57.06
		Sample	PIK3CA	YELLOW	51.47	0.063	25,447	964	24,483	59.29
A3	Post-S-LPS	Sample	PIK3CA	GREEN	353.7	0.026	25,219	5867	19,352	54.83
		Sample	PIK3CA	YELLOW	425.9	0.024	25,232	6889	18,343	45.9
B1	Post-chemotherapy	Sample	PIK3CA	GREEN	5.998	0.184	25,440	114	25,326	55.08
		Sample	PIK3CA	YELLOW	234.9	0.031	25,395	4096	21,299	56.1
B2	Post-IDS	Sample	PIK3CA	GREEN	7.29	0.169	25,478	135	25,343	50.04
		Sample	PIK3CA	YELLOW	127.1	0.041	25,479	2254	23,225	56.1
B3	8 months post-chemotherapy and IDS	Sample	PIK3CA	GREEN	3.405	0.245	25,470	64	25,406	57.38
		Sample	PIK3CA	YELLOW	22.94	0.095	25,470	428	25,042	53.55
C1	13 months post-chemotherapy and IDS	Sample	PIK3CA	GREEN	983.3	0.017	25,451	13,155	12,296	47.18
		Sample	PIK3CA	YELLOW	1357.3	0.016	25,451	16127	9324	55.46

Supplementary Material S3

Patient 3: A 56-year-old woman was diagnosed with advanced high-grade serous ovarian cancer, stage IIIC, in July 2020, after S-LPS surgery with drainage of 7 L of ascites and a finding of massive intraperitoneal dissemination of disease (PIV 10), for which upfront cytoreducibility was not achievable, and therefore a PDS was not performed. She underwent neoadjuvant chemotherapy with four cycles of Carboplatin AUC5 / Paclitaxel

175 mg/m2 every 3 weeks, with a partial response confirmed with a CT scan and serum markers.

Pre-chemo marker levels were elevated (CA125: 9797 mIU/mL, CA 19.9 966 mIU/mL—July 2020), with a clear reduction after NACT (CA125: 43.6 mIU/mL, CA 19.9 91.3 mIU/ mL—October 2020)

In November 2020, she underwent laparotomic IDS with radical hysterectomy, bilateral salpingo-oophorectomy, radical omentectomy, and pelvic peritonectomy, with no gross residual disease. She therefore completed adjuvant chemotherapy with four cycles of Carboplatin AUC5 / Paclitaxel 175 mg/m2 every 3 weeks, until January 2021.

During the follow-up, a slight increase in CA19.9 was observed starting from December 2022 (45.2 IU/mL --> 72.3 IU/mL February 2023) in the absence of a clinical or radiological confirmation of relapse. CA125 remained negative. The recurrence was identified radiologically following a PET-CT in April 2023 (two suspicious peritoneal nodules and one location in the right adrenal gland), 27 months after the last chemo.

In consideration of the platinum-free interval (PFI) > 12 months and the oligo-metastatic relapse, the patient underwent secondary LPS cytoreductive surgery, without macroscopic residual tumor in June 2023. She then underwent a second line of chemotherapy with carboplatin AUC 5 / Paclitaxel 175 mg/m2 (replaced by the III cycle with cisplatin 75 mg/m2 due to an allergic reaction to carboplatin).

From November 2023, she began maintenance therapy with Olaparib, a Poly ADP Ribose Polymerase (PARP1) inhibitor, and the therapy is still ongoing, currently with no recurrence of the disease.

Table S3. Absolute quantification of dPCR analysis for the plasma samples collected during the follow-up with the gDNA and tDNA for patient 3. The mutated probe of the specific mutation in the *CEP135* gene is indicated with yellow fluorescent dye and the wild type is marked with green dye.

Well	Sample/NTC/Control	Type	Reaction Mix	Channel (Name)	Conc. [cp/µL] (dPCR Reaction)	CI (95%) (dPCR Reaction)	Partitions (Valid)	Partitions (Positive)	Partitions (Negative)	Threshold
A1	gDNA	Sample	CEP135	GREEN	610.8	2.00%	25,235	9338	15,897	54.19
		Sample	CEP135	YELLOW	0.052	147.50%	25,503	1	25,502	62.73
A2	tDNA	Sample	CEP135	GREEN	324.6	2.70%	25,359	5482	19,877	48.45
		Sample	CEP135	YELLOW	147.5	3.80%	25,467	2668	22,799	58.65
A3	Pre-S-LPS	Sample	CEP135	GREEN	102.5	4.50%	25,383	1875	23,508	49.09
		Sample	CEP135	YELLOW	0.526	62.00%	25,383	10	25,373	44.88
B1	Post-S-LPS	Sample	CEP135	GREEN	57.14	6.00%	25,474	1067	24,407	49.09
		Sample	CEP135	YELLOW	0.157	106.40%	25,476	3	25,473	57.63
B2	Pre-IDS	Sample	CEP135	GREEN	2.157	31.00%	25,469	40	25,429	36.47
		Sample	CEP135	YELLOW	0	-	25,469	0	25,469	35.96
B3	Post-IDS	Sample	CEP135	GREEN	14.53	11.90%	25,472	272	25,200	51
		Sample	CEP135	YELLOW	0	-	25,472	0	25,472	42.33
C1	8 months post-chemotherapy	Sample	CEP135	GREEN	48.77	6.50%	25,456	902	24,554	56.1
		Sample	CEP135	YELLOW	0	-	25,458	0	25,458	42.33
C2	9 months post-chemotherapy	Sample	CEP135	GREEN	7.667	16.60%	25,475	139	25,336	52.91
		Sample	CEP135	YELLOW	0	-	25,475	0	25,475	43.61
C3	12 months post-chemotherapy	Sample	CEP135	GREEN	2.575	28.30%	25,478	48	25,430	35.51
		Sample	CEP135	YELLOW	0	-	25,478	0	25,478	41.06
D1	17 months post-chemotherapy	Sample	CEP135	GREEN	4.213	22.10%	25,489	79	25,410	54.83
		Sample	CEP135	YELLOW	0	-	25,490	0	25,490	37.23

D2	18 months post-chemotherapy	Sample	CEP135	GREEN	5.187	20.30%	25,472	93	25,379	45.9
		Sample	CEP135	YELLOW	0	-	25,477	0	25,477	39.78
D3	25 months post-chemotherapy	Sample	CEP135	GREEN	4.74	21.00%	25,482	87	25,395	47.81
		Sample	CEP135	YELLOW	0	-	25,484	0	25,484	41.06
E1	29 months post-chemotherapy	Sample	CEP135	GREEN	181.2	3.50%	25,449	3158	22,291	48.45
		Sample	CEP135	YELLOW	0	-	25,477	0	25,477	51.26

Table S4. Absolute quantification of dPCR analysis for the plasma samples collected during the follow-up with the gDNA and tDNA for patient 3. The mutated probe of the specific mutation in the *CCNF* gene is indicated with yellow fluorescent dye and the wild type is indicated with green dye.

Well	Sample/NTC/Control	Type	Reaction Mix	Channel (Name)	Conc. [cp/μL] (dPCR Reaction)	CI (95%) (dPCR Reaction)	Partitions (Valid)	Partitions (Positive)	Partitions (Negative)	Threshold
A1	gDNA	Sample	CCNF	GREEN	677.8	0.02	24,788	9945	14,843	77.78
		Sample	CCNF	YELLOW	0	-	25,420	0	25,420	75.48
A2	tDNA	Sample	CCNF	GREEN	318.1	0.027	25,307	5373	19,934	59.29
		Sample	CCNF	YELLOW	160.1	0.037	25,395	2875	22,520	51.64
A3	Pre-S-LPS	Sample	CCNF	GREEN	41.79	0.07	25,355	781	24,574	77.14
		Sample	CCNF	YELLOW	0.158	1.064	25,357	3	25,354	48.71
B1	Post-S-LPS	Sample	CCNF	GREEN	37.77	0.076	23,915	667	23,248	75.86
		Sample	CCNF	YELLOW	0.223	0.98	23,925	4	23,921	51.26
B2	Pre-IDS	Sample	CCNF	GREEN	0.486	0.653	25,425	9	25,416	42.33
		Sample	CCNF	YELLOW	0	-	25,425	0	25,425	35.96
B3	Post-IDS	Sample	CCNF	GREEN	4.063	0.225	25,351	76	25,275	63.43
		Sample	CCNF	YELLOW	0	-	25,352	0	25,352	39.78
C1	8 months post-chemotherapy	Sample	CCNF	GREEN	91.84	0.048	25,394	1668	23,726	70.13
		Sample	CCNF	YELLOW	0	-	25,400	0	25,400	46.16
C2	9 months post-chemotherapy	Sample	CCNF	GREEN	6.854	0.176	25,412	124	25,288	70.76
		Sample	CCNF	YELLOW	0	-	25,412	0	25,412	47.43
C3	12 months post-chemotherapy	Sample	CCNF	GREEN	1.175	0.428	24,410	21	24,389	56.36
		Sample	CCNF	YELLOW	0	-	24,410	0	24,410	35.96
D1	17 months post-chemotherapy	Sample	CCNF	GREEN	3.348	0.257	23,540	58	23,482	59.61
		Sample	CCNF	YELLOW	0	-	23,541	0	23,541	41.06
D2	18 months post-chemotherapy	Sample	CCNF	GREEN	1.123	0.438	25,269	20	25,249	43.61
		Sample	CCNF	YELLOW	0	-	25,269	0	25,269	41.06
D3	25 months post-chemotherapy	Sample	CCNF	GREEN	2.727	0.277	25,443	50	25,393	66.3
		Sample	CCNF	YELLOW	0	-	25,444	0	25,444	41.06
E1	29 months post-chemotherapy	Sample	CCNF	GREEN	81.29	0.055	22,298	1287	21,011	72.99
		Sample	CCNF	YELLOW	0	-	22,309	0	22,309	46.16

Supplementary Figures

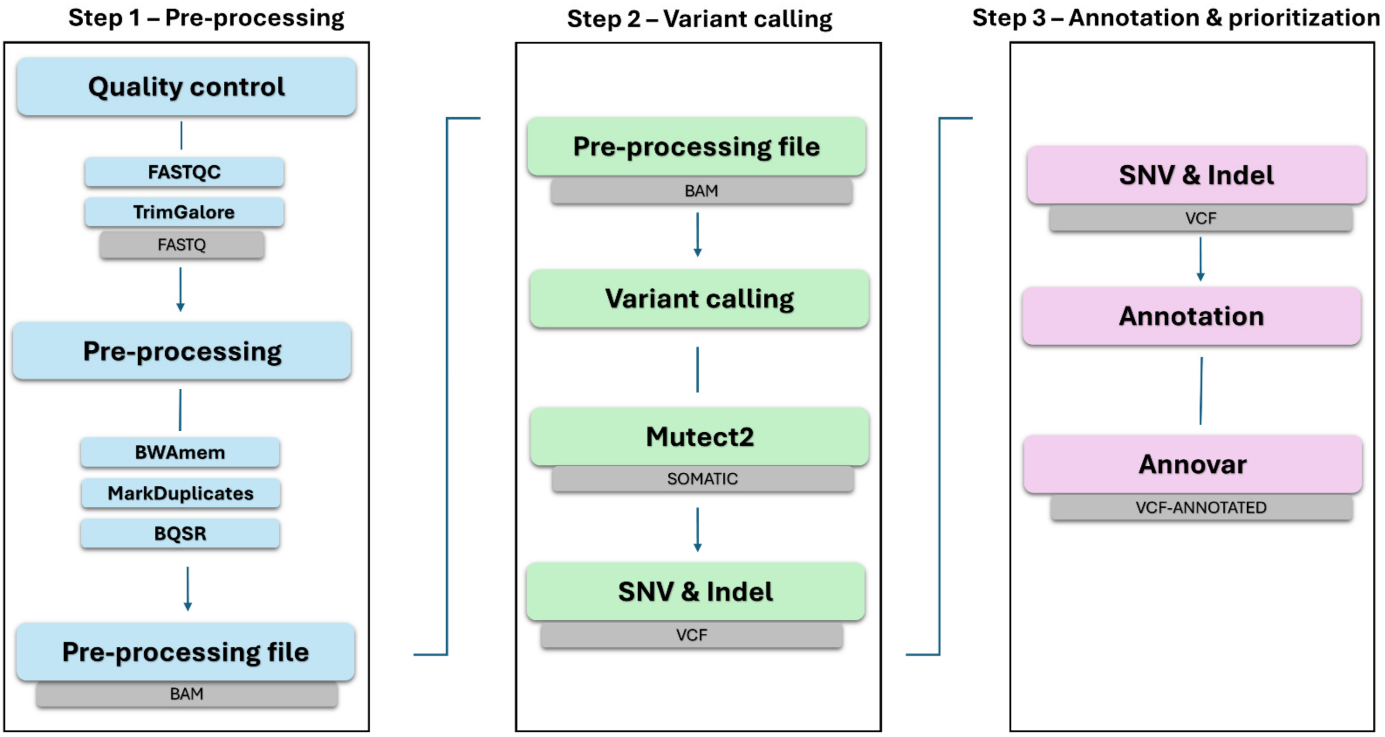

Figure S1. Schematization of bioinformatic analysis steps performed on raw WES data.

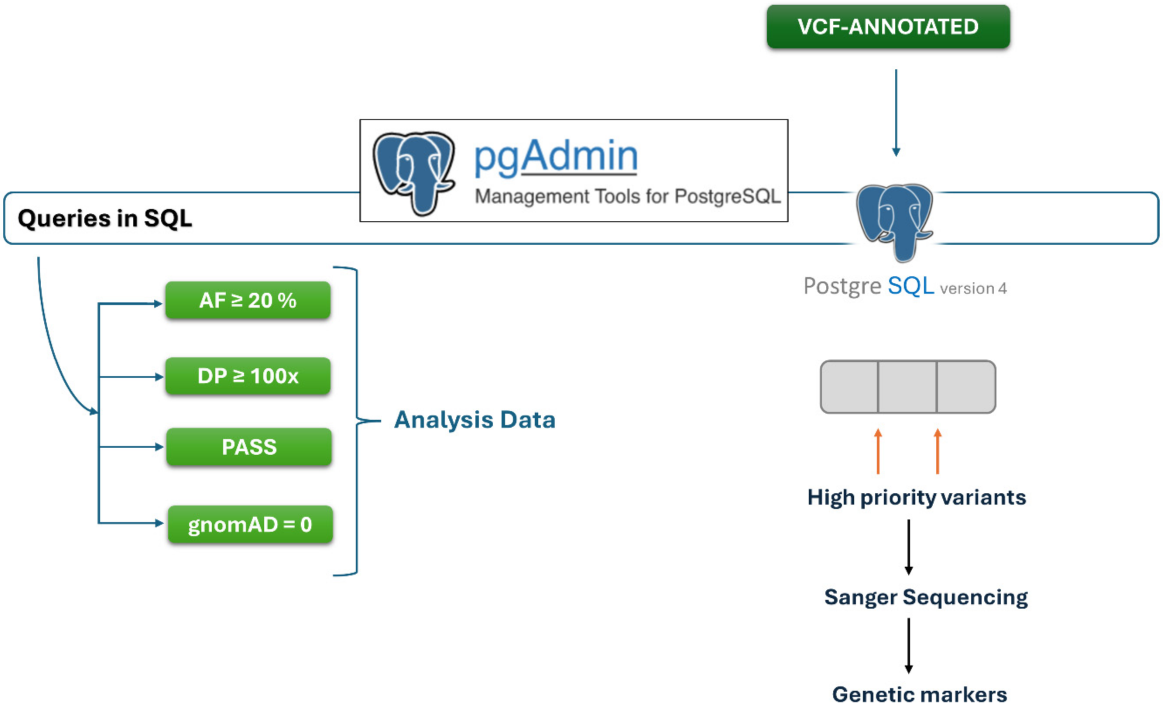

Figure S2. Clinical and mutations database. PASS: high probability true positive variants; AF: allele frequency; gnomAD: genome aggregation database; DP: deep coverage.
